# Clustering of atoms relative to vector space in the Z-matrix coordinate system and ‘graphical fingerprint’ analysis of 3D pharmacophore structure

**DOI:** 10.1007/s11030-023-10798-1

**Published:** 2024-01-28

**Authors:** Dilek Şeyma Kızılcan, Yahya Güzel, Burçin Türkmenoğlu

**Affiliations:** 1 Department of Chemistry, Faculty of Science, Erciyes University, Kayseri, Turkey; 2 Department of Analytical Chemistry, Faculty of Pharmacy, Erzincan Binali Yıldırım University, Erzincan, Turkey

**Keywords:** 3D QSAR, Dimensionality reduction, Pharmacophore, Clustering

## Abstract

The behavior of a molecule within its environment is governed by chemical fields present in 3D space. However, beyond local descriptors in 3D, the conformations a molecule assumes, and the resulting clusters also play a role in influencing structure–activity models. This study focuses on the clustering of atoms according to the vector space of four atoms aligned in the Z-Matrix Reference system for molecular similarity. Using 3D-QSAR analysis, it was aimed to determine the pharmacophore groups as interaction points in the binding region of the β2-adrenoceptor target of fenoterol stereoisomers. Different types of local reactive descriptors of ligands have been used to elucidate points of interaction with the target. Activity values for ligand-receptor interaction energy were determined using the Levenberg–Marquardt algorithm. Using the Molecular Comparative Electron Topology method, the 3D pharmacophore model (3D-PhaM) was obtained after aligning and superimposing the molecules and was further validated by the molecular docking method. Best guesses were calculated with a non-output validation (LOO-CV) method. Finally, the data were calculated using the ‘graphic fingerprint’ technique. Based on the eLKlopman (Electrostatic LUMO Klopman) descriptor, the Q^2^ value of this derivative set was calculated as 0.981 and the *R*^2^_ext_ value is calculated as 0.998.

## Introduction

The functional selectivity of β2-adrenoceptor (β2AR) was investigated using Fenoterol stereoisomers, which are believed to have therapeutic potential for asthma and congestive heart failure [1]. Fenoterol is a selective agonist for β2AR and has four stereoisomers due to the presence of two chirality centers. The stereochemistry of fenoterol also influences the binding of the receptor to different G proteins. Recent studies in the structural and biochemical fields have revealed that β2AR exists in multiple conformations and the number of conformations influences both its binding to cholesterol and signaling pathways. The MCET method was employed to investigate the local interactions between fenoterol stereoisomers and the β2AR receptor using clusters of atoms in 3D space. Datasets based on molecular similarities were organized and generated based on the 4-atom referenced vector space of the ZMR system. Clusters represent collections of atoms in the same region according to a given distance measure, forming the grouping process for 3D QSAR [2]. Clustering facilitates decision making when it comes to drug design because clusters are formed by visually aligning molecules to approximate the ligand binding geometry. 3D-QSAR models projected from clusters can explain the pharmacophore structure and show the quantitative relationship between 3D structural information (independent variables) and biological activity (response variable), representing an electronic map of the interface in the L-R interaction [3–5]. Accurate analysis of molecular activities depends on the perfection of pharmacophore revealed by the 3-dimensional similarities of molecules [6–8]. Therefore, molecular alignment methods, matching procedures of atomic stacks, and different-based overlapping molecule methods have been investigated to find a realistic pharmacophore in drug design [9, 10].

Over the last two decades, several overlapping methods have been developed and utilized for superposition, including Gaussian Volume Overlap [11], Volume Overlap Optimization [12], Field-Based [13], Distance-Based [14], Graph-Based [15], and Shape-Based [16]. These methods utilize different algorithms to form clusters for feature selection. The aim of feature selection is to reduce negative effects caused by high dimensionality, speed up the learning process, and improve generalization by reducing irrelevant and/or redundant features as much as possible. One such method is the clustering of atoms corresponding to the lattice points. Several methods, such as Molecular Shape Analysis, Receptor Surface Analysis, Weighted Holistic Invariant Molecular, Hypothetical Active Site Lattice, Comparative Field Molecular Analysis and Comparative Molecular Similarity Indices Analysis, arrange interaction regions in a vertical internal transformation with x, y, z coordinates [17–21]. In these methods, a molecule can be placed in a lattice structure with x, y, z Cartesian coordinates at regular intervals. The resulting lattice remains the same, and all molecules retain their original structure. This can lead to the formation of many clumps of atoms near certain points. The advantages of 3D modeling have been utilized in the structural method where clustering occurs, such as Non-Adjacent Atom Matching Structural Similarity, which can calculate the similarity score of atoms for different types of molecules [22]. Treating the atoms in a cluster as a single point helps to reduce data diversity by facilitating the processing of independent variables represented at a common point [23–25].

Our software, MCET, differs from other methods in that it clusters atoms based on their positions in the template structure rather than interacting network points [24, 26–29]. In this study, the results of the MCET program written by us on 26 compounds are presented.

## Material and methods

### Electron topological matrix (ETM)

One of the methods for identifying the pharmacophore is the electron topological matrix (ETM) approach. In this approach, the geometric and electronic properties of a molecule are defined within a matrix called ETM. Each conformer of each molecule is represented by an ETM. The diagonal elements of the ETM are an electronic value of the atom, and the non-diagonal elements are the geometrical distance or length value between atoms. It is information about the bond (bond order, Wieberg index, bond energy, etc.) for two chemically bonded atoms, or distance information for those that have not bonded. These common properties are represented by pharmacophore, determined from the electron topological submatrix of the activity. The results of pharmacophore affect the results of the calculated activity. The examination of the compound series, whose conformation analysis has been made, whose electronic structure has been calculated and whose experimental biological activity has been determined, by the Electron-Topological method, is as follows. First, the ETM or three-dimensional Electron Topological Matrix (3D-ETM) of each ligand is prepared. Since each ETM is symmetrical with respect to its diagonal elements (*a*^*a*^*), only the upper half of the matrix is shown in the figure. If the number of atoms in the molecule is n, the total number of independent elements is *n*(*n* + 1)/2. In 3D-ETM, the number of ETMs (*m*) depends on the selection of electronic parameters. Atomic parameters such as atomic charges, valence activities, polarizability and HOMO–LUMO energies that define the electronic properties of the molecule are selected as diagonal elements *a*^ (*i* = l, 2, 3…*n*, and *k* = l, 2, 3…*m*). Off-diagonal elements (*a*^) are of two types, a. If *i* and *j* represent two neighboring atoms bonded to each other by chemical bond, *a*^ can be one of the electronic parameters of the *i*–*j* bond such as polarizability, bond order and bond energy (total covalentionic). b. If *i* and *j* denote atoms that are not bonded to each other, then aj/^Rji^ denotes the distance between atoms. Thus, each matrix contains both the electronic (aij) and geometric parameter (Rij).

Although the geometric parameters are fixed for a given conformation of a molecule, electronic parameters are treated as different combinations of atomic and bond parameters. For example, different combinations such as bond lengths (bond parameters), atomic charges (atomic parameters) or atomic polarization-bond energy are examined as electronic parameters. Each of these combinations is effective in creating an ETM. If the number of combinations created for the electronic parameter is m and the number of molecules of the series examined is n, m ETMs are obtained for each molecule and n ETMs are obtained for each combination. After the ETM is created, the ETM elements of active compounds are compared one by one with the ETM elements of inactive ones to find a group of matrix elements that are present in active compounds but not in inactive compounds with a given degree of accuracy.

### APS and AG in MCET

To decide on APS and other AG, we need to examine the superposed structures of the active compounds. Pharmacophore contains a group of atoms necessary for activity. In addition to the atoms present in pharmacophore, the presence of atoms or groups of atoms in the existing molecular structure may have a decreasing or increasing effect on the activity. Some of the groups of atoms in the pharmacophore can increase hydrophobicity or form H bonds with the bioreceptor. These properties of these atoms are activity-enhancing properties, and this group of atoms is called AG atoms. Other group atoms, on the other hand, have a reducing effect on activity because they can create steric hindrance or protection during the interaction with the bioreceptor, and therefore such group atoms are called APS atoms. By adding the percent occurrence of each conformer, its energy, and temperature as a function, a formula for quantitative prediction of bioactivity is derived. MCET considers pharmacophore together with the parameters of the APS and AG groups. Thus, it transforms the idea of pharmacophore from a qualitative tool to a quantitative tool for bioactivity prediction.

### MCET method

To determine the structure–activity relationships of the compounds in the compound series; the created ETM matrices are read with the MCET program [30] and the group pharmacophore responsible for the activity is determined. The geometry of the template structure and the different positions of atoms in the five most active and least active molecules constitute sources of cluster points. With both geometric and electronic tolerance values for all molecules, similar atoms form clusters with a common core structure and serve to align with the template. The remaining oriented atoms form clusters in different regions on the scale of the maximum number of overlaps with the template according to geometric tolerance values.

The following should be considered when selecting the template structure:

- A compound with the most active and simplest structure that can represent those under investigation.
- A compound that is leading or commercial.
- A compound with the least number of functional groups.
- A rigid structure with a single conformer or a structure with the lowest energy in multiple conformers. [31–33].

The tolerance value may need to be adjusted to allow similar atoms of structurally similar molecules to cluster based on a common pattern. A tolerance value that is too large may result in unnecessary atoms being found in clusters, while a tolerance value that is too small may result in the absence of important atoms. In addition, the positions of core atoms in molecules can also pose challenges for clustering. These difficulties can result in a data set that does not adequately reflect spatial clustering characteristics [8]. To overcome these obstacles, it may be necessary to create alternative datasets with different numbers of atoms and clusters in different positions by adjusting the core structures and tolerance values. Considering the details of all locations in space, depending on the diversity of the molecular skeleton under consideration, can also help complete the dataset.

The three best-known structural arrangement systems for clustering in 3D space are:

(i) Internal molecular coordinates (bond lengths, bond angles and dihedral angles) [34],
(ii) A distance geometry descriptor (from a distance matrix and a four-atom reference point) [35].
(iii) Natural Cartesian coordinates [8, 36].

Each of these has different disadvantages:

- According to the atomic number in the internal coordinates, the bond length between two atoms is given by the bond angle formed by three atoms and the dihedral angle formed by four atoms (or two bonds) [37, 38]. It is necessary to avoid poor definitions of angles and dihedrals due to the linear arrangement of atoms in molecular coordinates [36].
- While it is powerful to distinguish a distance geometry with a four-point reference, the arrangement of these four points for all molecules requires a separate algorithm.
- Both Cartesian coordinates and z-matrix coordinates can give the distance between atoms in metric space. Accordingly, the structure of the molecule, the relative positions of the atoms, and the chirality of the asymmetric atoms in the molecule cannot be well defined in these arrangements. A structural alignment with a higher discriminating power is required, especially in structural or graphic and electronic matching approaches of stereo molecules whose biological activities are to be calculated [39, 40].

In this study, we utilized a structural arrangement called “z-matrix-reference” (ZMR) to distinguish stereo structures of molecules by combining both common and different features of the three structure arrangements. In the ZMR arrangement of the four atoms of the core structure according to Cartesian coordinates, the first atom is placed at the origin, the second atom on the z-axis (hence the name z matrix) and the third atom on the yz-plane. The fourth atoms are located in a region where the x, y, z coordinate signs are the same and form the starting positions of the molecules. The remaining atoms are oriented in vector space with respect to four-point references of similar location. Using ZMR, similarly arranged molecules can be analyzed for their local reactive effects, thereby developing quantitative conformation-activity relationships. To relate the similarities between structure and activity in stereo molecules, whose properties and biological effects are often significantly different, the interaction points of these molecules in 3D space must be located with the necessary differences [41, 42].

To accurately determine the similarity between stereo molecules, it is crucial to consider the important properties of atom positions and bonds. This requires consideration of atoms in vector space, a structural arrangement that allows easy and strong separation between stereoisomer structures [35]. In vector space, the position of each atom relative to the four atoms is defined by the x, y, and z coordinates. By providing these principles, it becomes possible to accurately compare the 3D structure of stereo molecules and determine their similarity, which is necessary to predict their properties and biological effects. After aligning the molecular structures in 3D using said new structural alignment, the resulting atomic stacks in similar regions can form clusters without the need for external knowledge. Atoms in the same cluster can interact similarly with the shared chemical domains of the virtual receptor, which is important in predicting the molecule's activity.

To reduce the complexity and dimensionality of molecular space, various dimensionality reduction (DR) approaches have been developed, which use linear and nonlinear vector spaces to transform high-dimensional data in QSAR studies [3, 43]. In these approaches, clusters can form vector spaces which are also referred to as “chemical property spaces” [44, 45]. The most common DR method used in these approaches is principal component analysis (PCA) [46], which treats the atomic stack as a single point in a cluster and converts it into data matrices as small-scale input. Other DR methods that are frequently used include principal coordinates analysis (PCooA) [47], Sammon mapping (SM) [48], Kernel PCA [49], Isomap [49], Autoencoders [50], t-Distributed Stochastic Neighbour Embedding (t-SNE) [51] and stochastic proximity embedding (SPE) [52]. The key feature in all these methods is the optimization of the DR guiding criterion, which is based on the geometric representation of data. The main objective of analyzing such geometric spaces is to discover relationships between the points in the complex data structure formed by the clusters [53].

A three-step reduction process is applied to transform the molecular structures into a graphical representation without losing data. First, the atoms arranged in the ZMR coordinate system were clustered according to their close neighborhoods, and the atoms in a common chemical area in each cluster are reduced to a single point. Second, vector space distances between atoms are calculated using such as x, y, z coordinates in the ZMR coordinate system and these distances are used to construct an electron topological matrix (ETM) representing different stereo structures. In one layer of the ETM, the distances between atoms in non-diagonal elements are given in Å, while the LRDs of atoms in diagonal elements exist electronically. The 3D ETM with the same distances and a different LRD in each layer is reduced to a 2D ETM where the distances and LRDs are represented in a single layer. Third, the interaction points in 3D space are reduced to a vector with consecutive number indices along an axis.

To simplify the visualization of non-bonding covalent and electrostatic interactions between ligands and receptors, a 2D graphical representation has been proposed. This representation shows how the activity changes at each interaction point and allows for the visualization of the increasing or decreasing effect of the auxiliary group (AG) or Anti-Pharmacophore Shield (APS) of the respective atom in a molecule. The quality and amount of AG or APS interaction at each point may vary from molecule to molecule. This approach, a new DR strategy, enables local quantitative interactions of molecules in three-dimensional space relative to the receptor, depending on LRDs, to be displayed in 2D graphics. The stereoisomers of fenoterol and their binding affinities to the β2-adrenergic receptor were taken from the literature to demonstrate the safety of DR strategies applied in stereo structures [54]. The theoretical results of the model obtained with 3D-QSAR in the MCET method are quite compatible with the experimental results. The skeletons of the molecules in Table 1 were drawn as using Spartan'08, and conformers were generated with the MMFF force field. To perform quantum chemical computations, the conformers were optimized with the Hartree–Fock functional method using the 6–31 G* basis set in water [55]. The resulting quantum information was recorded with the names of conformer files “n_c.txt” (n: molecule no, c: conformer no). Atomic charges, atomic coefficients, and interatomic distances of conformers were stored in the ‘etm.txt’ file in 2D ETM format, while Cartesian coordinate values were stored in the ‘koor.txt’ file. During model creation, all conformer information was taken from these two files as a data set.

**Table 1:** Stereoisomer structures, observed and predicted activity values of fenoterol compounds [56]

Molecule shape			Activity		Molecule shape			Activity	
			Obs	Pred				Obs	Pred
1		**R,R**	6.460	6.461	2		**S,S**	4.560	4.643
3		**R,S**	5.430	5.512	4		**S,R**	4.990	4.965
5		**R,R**	6.320	6.262	6		**S,S**	4.800	4.849
7		**R,S**	5.710	5.566	8		**S,R**	5.280	5.295
9		**R,R**	5.530	5.528	10		**S,S**	4.540	4.540
11		**R,S**	5.100	5.193	12		**S,R**	4.640	4.682
13		**R,R**	5.730	6.108	14		**S,S**	4.540	4.506
15		**R,S**	5.220	5.155	16		**S,R**	4.510	4.477
17		**R,R**	6.620	6.627	18		**S,S**	5.600	5.529
19		**R,S**	6.470	6.473	20		**S,R**	5.750	5.726
21		**R,R**	5.030	5.003	22		**S,S**	4.250	4.229
23		**R,S**	4.500	4.565	24		**S,R**	4.000	4.014
25		**R,R**	4.980	4.978	26		**S,S**	4.690	4.693

Models have been developed by applying the following methods within the scope of the MCET program with the fundamental quantum values of the molecules.

- To make the ligands compatible with the receptor, the molecules are positioned similarly to the atoms of the template.
- Molecules are aligned according to the core structures, and conformers that mediate the maximum number of atoms overlap have been selected to represent the molecular structure.
- The superposition of the molecule that allows maximum interaction with the receptor through each selected conformer is positioned.
- Various interaction fields have been established according to different LRDs used as electronic values of atoms.

Clusters consisting of atomic stacks that are similar and present in different regions of 3D space can have an enriched dataset due to various options. First, different chemical domains can be formed with different LRDs. Second, interaction fields can arise in different regions depending on different core structures. Third, clusters with the most mature and efficient atomic stacks can be formed by finding the optimum value of different tolerance values. Lastly, different sub-cluster scenarios can be created by selecting from the clusters using genetic algorithms (GA). All these options provide rich information that reveals various alternatives.

### Local reactive descriptors (LRDs) in MCET method

Given so many and different local interactions, it may be possible to obtain a true 3D QSAR study that will determine the best relationship between structural similarity and activity. For local geometric reactivity, the different electronic properties of atoms in clusters are due to four different classes of LRDs. In addition to the reference atoms in the template, atoms of some molecules are also used as references to form clusters in different regions in 3D space. Using tolerance values of less than one bond length, the formation of clusters with optimal atomic stacks is followed with statistical results. The considered sub-clusters are used as independent variables within the model. To reveal the framework of the study in more detail, the following 5 questions (**Q**s) need to be answered.

1. What is the advantage of aligning and superimposing the core structure with respect to the ZMR as the start of clustering?
2. To what extent do the clusters depend on the tolerance value that will result in an excellent chemical/structural information content?
3. What are the opportunities for 3D ETM implementation using four different classes of LRDs within MCET?
4. What are the ways to determine the optimum number of independent variables in pharmacophore formation?
5. What does it gain to explain the pharmacophore structure with a GF?

In order to find answers (**A**s) to these questions, the following objectives (**O**s) were tried to be achieved.

1. Aligning and superimposing the core structure with respect to the ZMR as the start of clustering allows for a consistent and standardized starting point for the analysis, which can reduce variability and increase the accuracy of the results [57].
2. The clusters depend on the tolerance value, and finding the optimum tolerance value is crucial to obtain clusters with the most mature and efficient atomic stacks. Tolerance values of less than one bond length are usually used to ensure the formation of clusters with optimal atomic stacks.
3. The four different classes of LRDs offer opportunities for 3D ETM implementation within MCET by providing a comprehensive description of the local electronic properties of atoms in clusters. This can enhance the accuracy and specificity of the analysis and lead to a more detailed understanding of the relationships between structural similarity and activity.
4. Determining the optimum number of independent variables in pharmacophore formation can be achieved using statistical methods such as partial least squares (PLS). These methods can help to identify the most significant independent variables and eliminate redundant or irrelevant variables, thereby simplifying the model and improving its predictive power.
5. Explaining the pharmacophore structure with a GF (grid-based force field) can provide insights into the energetics and interactions of the atomic stacks in the clusters. This can help to identify the key features that contribute to the activity and provide a basis for designing new molecules with improved properties.

1. In the ZMR coordinate system, the new coordinate values of the remaining atoms according to the arrangement of the atoms in the nucleus are applied systematically with similar translation, reflection, and rotation amounts:
  (I) While the coordinate values of the 1st functional atom (x, y, z) in the core structure are shifted to the origin (0_1_, 0_1_, 0_1_), all atoms are shifted similarly by the amount of the 1st atom. The coordinate matrix values of each atom are subtracted from the previous values of the first atom and calculated as the coordinate values of the new position after the translation (*x*_*n*_^*p*^, = *x*_*n*_^*p*′^ − *x*_*n*_^1^; y_n_^p^ = y_n_^p′^—y_n_^1^; z_n_^p^ = z_n_^p′^ − z_n_^1^; p′ and p = 1,2,3…P_n_), where n: molecule number; x, y, and z: coordinate values; *p*′ and *p*: show the previous and next position values, and *P*_*n*_: the total number of atoms in the n-molecule. 1: Represents the 1st atom and taking *p* and *p*′ = 1 means that the new *p* position is pulled to the origin. Coordinate values of the nth molecule at the p-position are denoted by (0_1_, 0_1_, 0_1_) as *x*_*n*_^*p*^, = 0, *y*_*n*_^*p*^ = 0, and *z*_*n*_^*p*^ = 0.
  (II) In order for the 2nd atom to come to the z-axis (0_2_, 0_2_, z_2_) after the first operation, all atoms are rotated on the x and y axes, similar to the 2nd atom, by the φ_x_ and θ_y_ angles of the 2nd atom, respectively (Fig. 1). Here, the angles φ_x_ and θ_y_ are the angles from the projection of the 2nd atom to the z-axis with respect to the yz-, xz-planes, respectively.

**Fig. 1:**
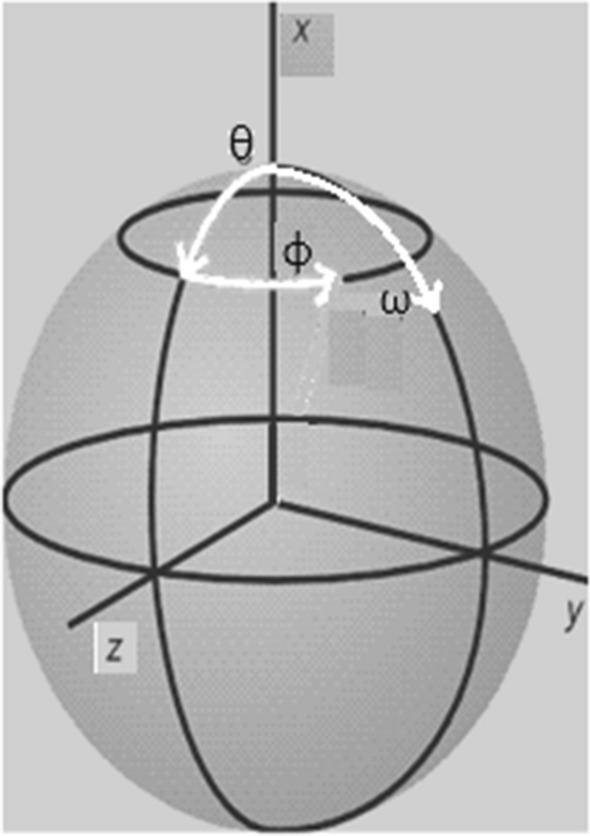
Rotation angles in x, y, z-axis is given by θ φ ω
  (III) After the 1st atom is placed at the origin and the 2nd atom is placed on the z-axis, the 3rd atom is arranged in the yz-plane so that x_3_ = 0 (0_3_, y_3_, z_3_). To do this, all atoms are rotated around the z-axis by the angle ω_z_ to the y-axis with respect to the projection of the 3rd atom on the xy-plane. ZMR employs simple translations and rotations, with the same transformation coefficients as described above, to be applied to all atoms of the same scale without altering their internal coordinate positions relative to each other. In the ZMR approach, it is expected that the 4th atom of the core structure will be in the coordinate region of the same sign (x, y, z) and approximately the same value for all molecules. This ensures that bad definitions of angles and dihedral angles in internal coordinates can be avoided, thanks to the arrangement of the Cartesian coordinates of the atoms based on the four atoms. Only atoms that overlap with their vector space distance values relative to the four atoms in the core structure are included in the system, leading to the formation of clusters based on the atomic location of all molecules.
    The positions of the molecules being investigated may either match those of the template or be different. If a molecule's atom in a different position can serve as a new reference atom, it can be added to the total number of reference atoms. The positions of these atoms are called 'reference atoms' as they lead to the formation of new datasets in addition to those of the template. The number of reference atoms needed to detect all interactions between L-R depends on the diversity of molecules forming clusters at different positions [58]. Local interactions in 3D space were investigated according to the clustering formed at the rate of the molecular diversity under investigation. Each molecule contains at most one atom in a cluster. The number of molecules in the clusters in the core structure is the same and is equal to the total number of molecules (N). Not all molecules in any of the other clusters contain atoms. Molecules containing atoms from one cluster to another, and their numbers, are often different. Therefore, the maximum number of molecules containing atoms in some clusters is *N*′ ≤ *N*. If the total number of atoms in the *p*^th^ cluster from the total P cluster is given as Ap, then only one atom (A_n_^p^) of the n^th^-molecule exists in the *p*^th^ cluster, {a_1_^p^, a_2_^p^,…a_n_^p^, …,a_N_^'p^; *n* = 1, 2…*N*′} [59]. The position of the reference atom in the p-cluster is represented as p ∈ ℝ^3^ with x, y, z-coordinate values. Even if an atom is only similar in terms of local geometric values without electronic similarity, it can be placed in the same cluster and added as a candidate atom ap to the atomic sequence within the cluster. Considering a cluster as a point, the number of atomic arrays N' in the cluster can be reduced to a single point, so that ℝ^3^*N*′_p_ → ℝ^3^_p_. Different or identical atoms in each cluster can be represented by groups such as A^1^, A^2^, A^P^ for the total number of clusters P. For each p-cluster’s reference atom a_ℓ_^p^, the coordinate value ℝ^3^ is known and represents the point position of the cluster. The atoms of different molecules within this p-cluster can be represented by A^p^ = {a_1_^p^(M_1_), a_2_^p^(M_2_),…a_n_^p^(M_n_),…a_N_^p^(M_N_)}. We can determine which clusters contain atoms of a molecule, just as we can identify which molecules contain atoms in a cluster. Each atom in the p-cluster has two properties: its geometric property, represented by its atomic coordinates (a_n_^p^(M_n_) ∈ ℝ^3^), and its electronic property, which is one of four different classes of LRDs (a_n_^q^(M_n_) ∈ ℝ^4^). Atoms of a molecule can have as much influence as their own amount of LRD in the p-cluster to which they belong.
    The core structure with at least one functional atom, such as N, O or S, is first derived from combinations of atoms in the template. This structure is considered provided that all active molecules are present in at least one conformer. The conformation of a molecule is chosen by looking at the maximum number of atoms overlapping the template. Depending on the structure chosen, clusters from other overlapping atoms are added to the core structure that forms the basis of the cluster. Clusters are located at a distance greater than one bond length from each other. Considering that some of the clusters may correspond to interaction points, different sub-clusters are suggested. Among these sub-clusters, one of the most coherent is considered as pharmacophore in 3-dimensional space. Both the geometric positions and electronic values at each point of the core structure in the sub-cluster may be approximately the same in all molecules, resulting in the same changes in activity [22]. Atoms in the remaining elements of the sub-cluster can have quite different electronic values, resulting in different activity values for each molecule. In fact, if some molecules do not contain atoms in these elements of the sub-cluster, no change in the activity of the molecule is observed. Depending on whether there are atoms in the sub-cluster and the number of electronic values of the atoms, changes occur in the activity of a molecule.
2. In addition to the arrangement of atoms in the cluster, another important factor limiting the development of the pharmacophore model is tolerance values. In the core structure, the atoms are aligned with both electronic and geometric tolerance values, while the remaining atoms are superimposed by geometric tolerance only. The number of atoms in the cluster depends on the varying geometric and/or electronic tolerance scale. If atoms cannot be placed in the cluster or are placed unnecessarily due to small or large tolerance values, respectively, it can affect the evolution of the model. Clustering is achieved with an electronic similarity tolerance of 10–20% and a geometric similarity tolerance within a cube volume (dτ = dxdydz) close to the typical bond length (ε = dx = dy = dz =  ~ 1.0 Å). To create different tolerance values, 0.2% and 5% increments are used for both geometric and electronic properties. The activity of the molecule can vary depending on the tolerance value and the presence or absence of atoms in a cluster. To mature clusters, different tolerance values are used to provide input and output to the cluster according to the neighborhood of the atoms. The improvement or deterioration of the cluster is controlled by adding new atoms to the cluster after the tolerance value is increased, typically 0.5 to 1.5 Å. While determining the sub-cluster, the change in the correlation coefficient (*R*^2^) is monitored and it is determined whether a new addition is needed. Sub-cluster elements with the highest *R*^2^ value are kept and it is determined whether new elements need to be added. When the increase in the number of correlations is insignificant (approximately 0.5–1% increase), the addition of the number of independent variables pharmacophore is stopped. As a result, the sub-cluster that gives the best statistical result and has the least number of elements can be selected. An atom in the proposed sub-cluster contributes to the interaction of its molecule in the cluster region. The activity of its molecule can vary depending on whether it has an atom in an element of the sub-cluster and whether the electronic value of the atom has positive (or negative) and small (or large) contributions.
3. 2D ETM is a more practical option than a 3D ETM. In the 2D ETM format, the interatomic distances arranged according to ZMR coordinate values and the LRD values of four different atom classes are organized into an important dataset. Although the 2D ETM values of a conformer contain different LRDs, the interatomic distances are represented by a fixed geometry and remain the same. Organized according to ZMR coordinate values, 2D ETM gives a distinctive feature to stereo structures. The pharmacophore model changes as the LRD changes in a 2D ETM due to both the parameter values on the receiving side and the positions of the sub-clusters that make up the model. The chemical domain type and parameter size of the receptor side corresponding to the covalent and/or electrostatic value of the LRD are shown in Table 3. Both the LRD argument and the corresponding parameters are included in the pharmacophore model [60, 61].
  Atomic partial charges in a molecule only cause electrostatic interactions, while atomic coefficients in the Fukui (f(r), f^+^(r) and f^−^(r)) indices and in the boundary orbitals cause nonbonded covalent interactions. Typically, neither covalent nor electrostatic interactions alone are sufficient. Atomic coefficients allow for non-covalent interactions, while ionic (‘ ± ’) and van der Waals interactions take place on atomic charges. The Klopman Index (KI) is an important property that characterizes the diversified ionic and non-covalent interactions of an atom simultaneously with a single index. Both the Fukui Index and the KI have Hard-Soft Acid–Base (HSAB) properties [62].
  Equation (1) provides a simplified version of the KI, where two terms represent the two types of interactions. Different LRDs of the KI can be obtained by combining different species in these two terms. The first term on the right side of the Eq. (1) represents hard interactions, while the second term represents soft interactions. Various combinations are possible between the atomic charges in the first term (natural, Mulliken and electrostatic) and the atomic coefficients in the HOMO (or LUMO)-Frontier orbital in the second term.
  1 $$\Delta E=\frac{{Q}_{nuc}{Q}_{elec}}{4\pi \varepsilon R}-\frac{{2\left({c}_{nuc}{c}_{elec}\beta \right)}^{2}}{{E}_{HOMO\left(nuc\right)}-{E}_{LUMO\left(elec\right)}}$$
  In the Eq. (1), the symbol Q represents the atomic charge, ε represents the permeability, R represents the distance between two atoms in the L-R, c represents the atomic coefficient in the boundary orbital which can act as a nucleophile or electrophile, β represents the resonance integral, and E represents the energy level of the boundary orbital.
  2D ETM can handle electrostatic and non-bonding covalent interactions between two molecules as nucleophilic/electrophilic behaviors in various ways. Different data sets with different LRD values are expected to have different contributions to the dependent variables of molecules [44, 45, 63] (Table 2).

**Table 2:** Parameter size of the receptor side chemical domain for four different LRDs on the ligand side

Units of the ligand side				Parameter size of the receptor side	
*k ***= **	LRD class	Electrostatic/covalent interaction	Descriptor property	Kappa (*κ*)	Xi (*ξ*)
1	Partial charge	Natural	Q_nuc(elec)_	$\frac{{Q}_{elec(nuc)}}{4\pi \varepsilon r}$	–
		Mulliken			
		Elektrostatic			
2	Local atomic Fukui index	Electrophilic attack (f^−^(r))	${{\rho }_{HOMO\left(r\right)}\approx f}^{-}(r)=({\frac{{\partial \rho }^{-}(r)}{\partial N})}_{V}$	–	${{\rho }_{LUMO\left(r\right)}\approx f}^{+}(r)=({\frac{{\partial \rho }^{+}\left(r\right)}{\partial N})}_{V}$
		Nucleophilic attack (f^+^(r))	${{\rho }_{LUMO\left(r\right)}\approx f}^{+}(r)=({\frac{{\partial \rho }^{+}\left(r\right)}{\partial N})}_{V}$	–	${{\rho }_{HOMO\left(r\right)}\approx f}^{-}(r)=({\frac{{\partial \rho }^{-}(r)}{\partial N})}_{V}$
		Radical attack	${f}^{\pm }(r)=1/2[{\rho }^{+}\left(r\right)+ {\rho }^{-}\left(r\right)]$	–	${f}^{\pm }(r)=1/2[{\rho }^{+}\left(r\right)+ {\rho }^{-}\left(r\right)]$
3	Frontier orbital	HOMO	c^2^_nuc HOMO_	–	$\frac{{2\left({c}_{elec}\beta \right)}^{2}}{{E}_{HOMO\left(nuc\right)}-{E}_{LUMO\left(elec\right)}}$
		LUMO	c^2^_elec LUMO_	–	$\frac{{2\left({c}_{nuc}\beta \right)}^{2}}{{E}_{HOMO\left(nuc\right)}-{E}_{LUMO\left(elec\right)}}$
4	Klopman index	HOMO&charge	c^2^_nuc_, Q_nuc_	$\frac{{Q}_{elec}}{4\pi \varepsilon R}$	$-\frac{{2\left({c}_{elec}\beta \right)}^{2}}{{E}_{HOMO\left(nuc\right)}-{E}_{LUMO\left(elec\right)}}$
		LUMO&charge	c^2^_elec_,Q_elec_	$\frac{{Q}_{nuc}}{4\pi \varepsilon R}$	$-\frac{{2\left({c}_{nuc}\beta \right)}^{2}}{{E}_{HOMO\left(nuc\right)}-{E}_{LUMO\left(elec\right)}}$
  The positions of atoms in an M-molecule are calculated based on their coordinate values in 3D space. The atoms are grouped into P clusters, and each cluster is represented by a different index, p. The x, y, z values of each atom are then converted into distances in the ETM matrix, resulting in a reduction from ℝ^3^ to ℝ. The electronic values, qk, for atoms with positions up to P in a molecule are found in the diagonal elements of the ETM with the same index. The distance between atoms at positions pi and pj is calculated in Å as *d*_*i,j*_ =|*pi* − *pj*|, where *i* ≠ *j*. The atomic numbers of the nth molecule are placed in the row and column atomic index numbers of *i* and *j*, forming a layer 1 matrix in 2D ETM. There are non-diagonal elements of the ETM in the number {*P*(*P* − 1)/2} between the *P* atoms. Since the distance values in row-column numbers that are symmetrical to the diagonal axis of the matrix are the same (for example, the distance between atoms 1 and 3 d_1,3_ = d_3,1_), only the upper triangle matrix of the ETM is used. New additional layers (*k* > 1) form the 3D ETM as the diagonal (*i* = *j*) values for different LRDs change, while the non-diagonal values remain unchanged. For practicality and ease of understanding, the 3D ETM is simplified to a 2D ETM by moving only the diagonal elements to the upper rows of the ETM according to the same atomic index, while keeping the non-diagonal elements the same. This results in a dimension reduction without any loss of data (see Fig. 2).

**Fig. 2:**
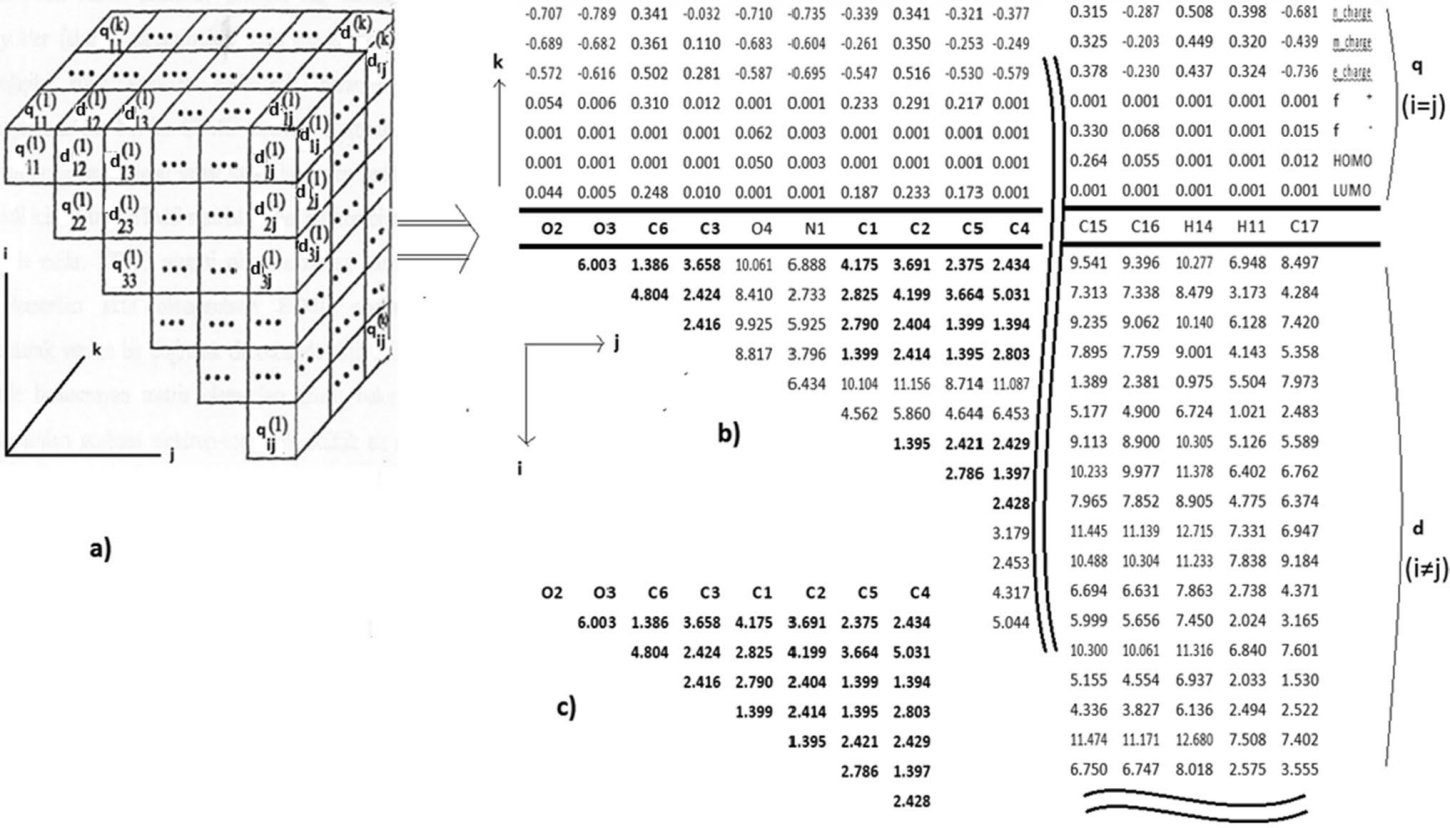
**a** 3D ETM; LRDs of diagonal elements (*q*_*i*_) and distances between non-diagonal elements (*d*_*i,j*_) are shown. **b** Different LRDs are given in rows in ETM. **c** Electron topological sub-matrix (ETSM) in pharmacophore’s ETM is marked in bold
  The local p-position of atoms in each conformer was determined by recording geometric distances (*d*_*i,j*_) and electronic values (*q*_*i*_) in files named ‘ETM.txt’, and x, y, z coordinate values in the ZMR system in files named ‘Z-matrixCoord.txt’. The order of atoms in both files is the same, with the core atoms (four atoms) that make up the vector space placed in the first rows. Although the ETM and Cartesian coordinate values are rearranged for each held core structure, the dataset remains fixed since the position values of atoms in the conformer relative to the first four atoms are in the vector space [64].
  The ETM shown in Fig. 2 is a 2D representation of a 3D matrix. For each different LRD value, a one-layer ETM is formed, which then combines to form a 3D ETM. The 2D ETM is created by moving only the diagonal elements to the upper rows of the ETM according to the same atomic index, while keeping the non-diagonal elements the same.) [59, 64–66].
  An electron topological sub-matrix (ETSM) is a sub-cluster of the ETM that includes a specific set of atoms within the molecule. The ETSM is obtained by selecting a group of atoms and taking the corresponding sub-matrix of the ETM. The ETSM contains information about the electronic properties and interatomic distances of the selected atoms and can be used to analyze local electronic properties of the molecule. The ETSM is shown in bold within the ETM in Fig. 2.
  The pharmacophore structure of a molecule is defined by the ETSM values, which play a crucial role in determining its activity and are extracted from the ETM of a selected conformer of the molecule. Although there may be slight variations, the ETSM values for a given molecule remain constant within a certain tolerance range. However, the number of atoms in the ETSM may differ between molecules. The ETSM of a molecule is derived from the geometric and electronic properties of the atoms that occupy the pharmacophore structure.
  To determine the actual interaction between two molecules (L-R), it is important to identify the LRD that gave rise to the chemical field. Therefore, choosing the appropriate LRD from the 2D ETM is crucial, as well as constructing the clusters. From each of the four different classes of LRD given in Table 2, a final model can be proposed as the pharmacophore construct. During the processing of each LRD, the molecules are realigned in the ZMR system according to the new core structures derived from the template. For each LRD, the process of organizing clusters, creating sub-clusters, estimating parameters, and calculating activities is repeated, and statistical results are stored for comparison with others.
4. To identify the optimal data set and independent variables, a genetic algorithm (GA) was employed. GA has been demonstrated to produce reliable and precise predictions in QSAR modeling in recent studies [67]. The GA generates a population of ‘chromosomes’ through random crossover and mutation operations, and the fitness function is used to evaluate them. Within the GA, independent variable selection and size reduction, model optimization, conformational search, insertion, and variation analysis were all conducted.
  The Levenberg–Marquardt algorithm was used to calculate the parameters of the corresponding spots on the receptor side for a selected sub-cluster. The relationship between the energy values resulting from the interaction of these corresponding points on the L-R sides and the activity is described by the nonlinear Eq. (2). The results were evaluated using the PLS, which considers the differences between the theoretical and experimental activities calculated using Eq. (2). The PLS involves expressing the sum of the squares of a set of activity errors with the model function of the sub-cluster. In this way, a mathematical model parameterized according to sub-clusters was obtained through the training and external test sets, with the goal of minimizing errors in theoretical and experimental activities. The model was validated using the Leave One Out-Cross Validation (LOO-CV) approach on the training set and then tested on the external test set.
  2 $${A}_{n}={A}_{l}{e}^{-(\Delta {E}_{n}-\Delta {E}_{l})/RT}$$
  where *A*: activity value, *n* and *ℓ*: number of studied and reference molecules, respectively, Δ*E*: binding energy in Joules arising from interaction points between L-R, *R*: Ideal gas constant in 8.314 J/mol-K, *T*: Body temperature is 310 K.
  To optimize the nonlinear system of equations, the Levenberg–Marquardt algorithm is employed, which employs a non-monotonic technique to achieve convergence [68]. The Levenberg–Marquardt algorithm involves two numerical minimization procedures, namely the gradient descent method and the Gauss–Newton method. In the gradient descent method, the parameters are updated in the direction of steepest descent to minimize the sum of the squared errors. On the other hand, the Gauss–Newton method assumes that the least square’s function is locally quadratic in the parameters, and it finds the minimum of this quadratic function to minimize the sum of squared errors.
  The four processes considered above are repeated for each new core structure, arranging the molecules in the z-matrix coordinate, forming clusters, sub-clusters, and calculating pharmacophore structures with different LRD classes.
5. The clusters in the 3D coordinate system cannot represent the activity as the 4th dimension on the graph. Instead, it is more practical to display the change in the dependent variable (activity) on the y-axis versus the change in the x-axis, where the index numbers of the independent variables are given. This allows for easy visualization of the interaction amount of pharmacophore with a GF. It is noteworthy that this simple and understandable application is, to the best of our knowledge, the first to demonstrate the interaction between L-R. By reducing the vector values of the independent variables in 3D to a 1D index and GF, we can show the activity change at each point without any loss of information, which adds value to the analysis.

## Results and discussion

This study aimed to develop and validate a model using 26 fenoterol analogs as potential selective and potent β2-AR agonists in MCET. Multiple pharmacophore models were created using various LRDs from four LRD classes, with a perfect sub-cluster identified by adding new clusters to a new core structure. The activity values of each fenoterol structure were tracked to develop a model using different LRD datasets in clusters. The models were trained and validated on 21 compounds using LOO-CV and the results were predicted on an external test set of only 5 compounds. The best model was determined based on high-statistical performance of Q^2^ for the training set and R^2^_ext_ for the external test set using LOO-CV. The table in the paper shows the results of different LRD classes with high values in both training and test sets (Table 3).

**Table 3:** *Q*^2^ and *R*^2^_ext_ values calculated with different descriptors of the ligand side for fenoterol stereoisomers

Descriptor	*Q*^2^	*R*^2^_ext_
n_Charge	0.751	0.882
f^−^_Fukui	0.890	0.850
LUMO	0.887	0.843
eLKlopman	**0.981**	**0.998**

The statistical results of one type of LRD that stands out among the four classes of LRD are given in Table 3. For example, here, eLKlopman means that the Klopman Index, *e*: Electrostatic atomic charges for electrostatic interactions and *L*: atomic coefficients of Lumo on the ligand. Similarly, n_Charge: Among the charges, Natural charge means that there is a type of LRD that stands out more than Mulliken and electrostatic charges. The KI, which has the best values (*Q*^2^ = 0.981 and *R*^2^_ext_ = 0.998) among the LRDs, is compared with the most recently published study in Table 4 [56]. The root mean square of error (RMSE) in the training and test sets, together with the F-test, are given as 0.099, 0.024, and 3.398, respectively. We can see that the F statistic (3.398) is larger than the F critical one tail (2.866), so we will reject the null hypothesis.

**Table 4:** Comparison of the observed values of β2-AR binding affinity^①^ with predictions made by CoMFA^②^ and MCET methods^③^ in two different 3D-QSAR models

Num	Comp	Obs.^①^	Pred.^②^	Res.^②^	Pred.^③^	Res.^③^
N01	R,R-1	6.460	6.127	0.333	6.461	− 0.001
N02	S,S-1	4.560	4.784	− 0.224	4.643	− 0.083
N03	R,S-1	5.430	5.380	0.050	5.512	− 0.082
N04	S,R-1	4.990	5.052	− 0.062	4.965	0.025
N05	R,R-2	6.320	6.354	− 0.034	6.262	0.058
N06	S,S-2	4.800	5.033	− 0.233	4.849	− 0.049
N07	R,S-2	5.710	5.629	0.081	5.566	0.144
N08	S,R-2	5.280	5.281	− 0.001	5.295	− 0.015
*N09	R,R-3	5.530	5.774	− 0.244	5.528	0.002
N10	S,S-3	4.540	4.496	0.044	4.540	0
N11	R,S-3	5.100	5.093	0.007	5.193	− 0.093
N12	S,R-3	4.640	4.697	− 0.057	4.682	− 0.042
N13	R,R-4	5.730	5.764	− 0.034	6.108	− 0.378
*N14	S,S-4	4.540	4.435	0.105	4.506	0.034
N15	R,S-4	5.220	5.033	0.187	5.155	0.065
*N16	S,R-4	4.510	4.690	− 0.180	4.477	0.033
N17	R,R-5	6.620	6.787	− 0.167	6.627	− 0.007
N18	S,S-5	5.600	5.520	0.080	5.529	0.071
N19	R,S-5	6.470	6.129	0.341	6.473	− 0.003
N20	S,R-5	5.750	5.745	0.005	5.726	0.024
*N21	R,R-6	5.030	5.189	− 0.159	5.003	0.027
N22	S,S-6	4.250	4.062	0.188	4.229	0.021
N23	R,S-6	4.500	4.380	0.120	4.565	− 0.065
N24	S,R-6	4.000	4.061	− 0.061	4.014	− 0.014
*N25	R-7	4.980	5.223	− 0.243	4.978	0.002
N26	S-7	4.690	4.531	0.159	4.693	− 0.003

*Test set molecules

Observed β2-AR binding affinity values^①^ are taken from the literature [54]. For 3D-QSAR models, the predicted values from the literature^②^ [56] and the MCET method^③^ are given.

The developed model in this study utilizes the KI, a class of LRD that includes both electrostatic and covalent descriptors and features HSAB principles. Atomic partial charges are calculated based on the coefficients of the respective atom in the occupied orbitals, while the coefficients of the atoms are taken from the wave functions of the molecules' HOMO/LUMO. The KI is formed by combining the values from both terms on the right side of Eq. (1), with a small HOMO–LUMO gap indicating a predominance of covalent interactions, while a large gap indicates a predominance of electrostatic interactions. The QSAR model obtained with the KI considers both electrostatic and covalent interactions and allows for the calculation of receptor-side parameters *ĸ* and *ξ*, as shown in Table 5. The Levenberg–Marquardt algorithm is used to consider the parameters of the interaction point simultaneously for both terms of KI in MCET. [26, 69, 70].

**Table 5:** Atomic positions, Cartesian coordinates, *ĸ* and *ξ* values of reference molecules (n01 and n24) in the series of fenoterol stereoisomers

Molecule No	Atom No	*X*	*Y*	*Z*	Position	*ĸ* value	*ξ* value
**n01**	**O2**^**a**^	**0**	**0**	**0**	1	**5.366**	**− 8.661**
**n01**	**O3**^**b**^	**6.003**	**0**	**0**	2	**− 1.064**	**− 4.781**
**n01**	**C6**^**c**^	**1.239**	**0.621**	**0**	3	**11.186**	**0.728**
**n24**	**C3**^**d**^	**3.630**	**0.377**	**− 0.262**	4	**− 1.644**	**27.994**
n24	C1	3.792	1.746	**− **0.017	5	**− **1.965	8.063
n24	C2	2.671	2.536	0.238	6	5.450	43.398
**n24**	**C5**	**2.355**	**− 0.188**	**− 0.250**	7	**2.574**	**− 63.367**
n24	H1	3.705	4.151	0.457	8	0.454	26.256
n24	C7	4.825	**− **0.483	**− **0.620	9	0.678	8.425
n24	C8	5.047	**− **0.509	**− **2.152	10	0.084	2.688
n24	C4	1.388	1.983	0.245	11	6.237	19.930

^a, b, c^ and ^d^The first four atoms in the core structure

**Fig. 3:**
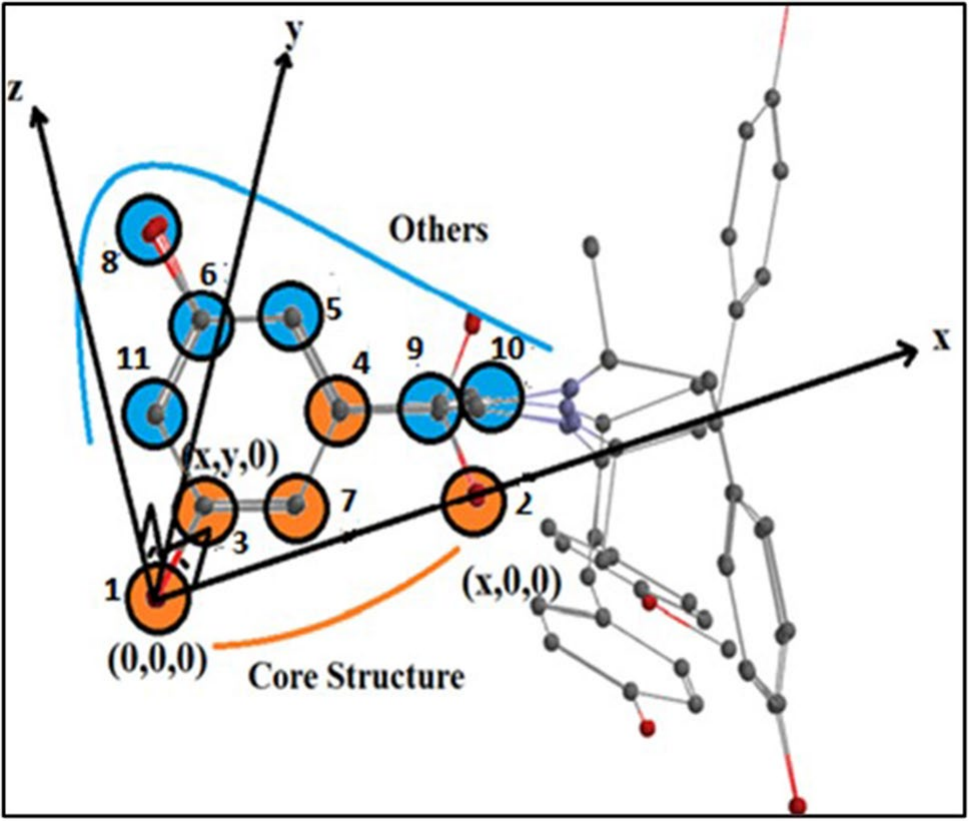
Representation of pharmacophore with the placement of the core structure in the Z-matrix coordinate system

**Fig. 4:**
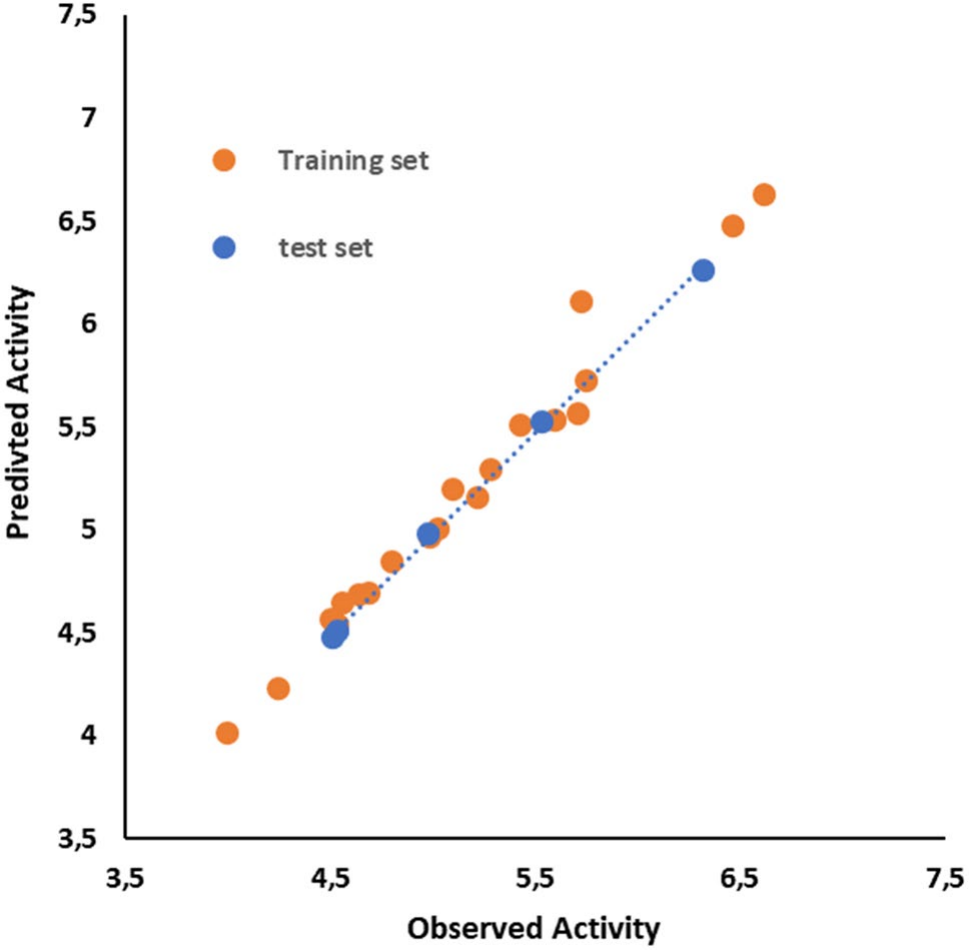
Experimental and calculated activity plot of the training and test sets of fenoterol stereoisomers

Positions marked as 1, 2, 3…,11 whose coordinates and parameters are given in Table 4, and the layout of the pharmacophore structure consisting of P = 11 interaction points in the Z-matrix coordinate system are presented in Fig. 3. Figure 4 shows the congruence of observed and predicted activity.

The study utilized 11 interaction points on the active side of the receptor, labeled as 1, 2, 3… and 11, to calculate interaction energies between ligand atoms and receptor parameters using Eq. (1). Subsequently, activity values were computed using Eq. (2), based on these interaction energies. The resulting activity changes for each interaction point were referred to as GF and plotted on the y-axis against the interaction point number on the x-axis. While the activity changes were visualized in 3D space with various shapes and visuals, it was crucial to present GF in a simple two-dimensional graphic as it captures the activity change in two dimensions without the need for a 4th dimension.

**Fig. 5:**
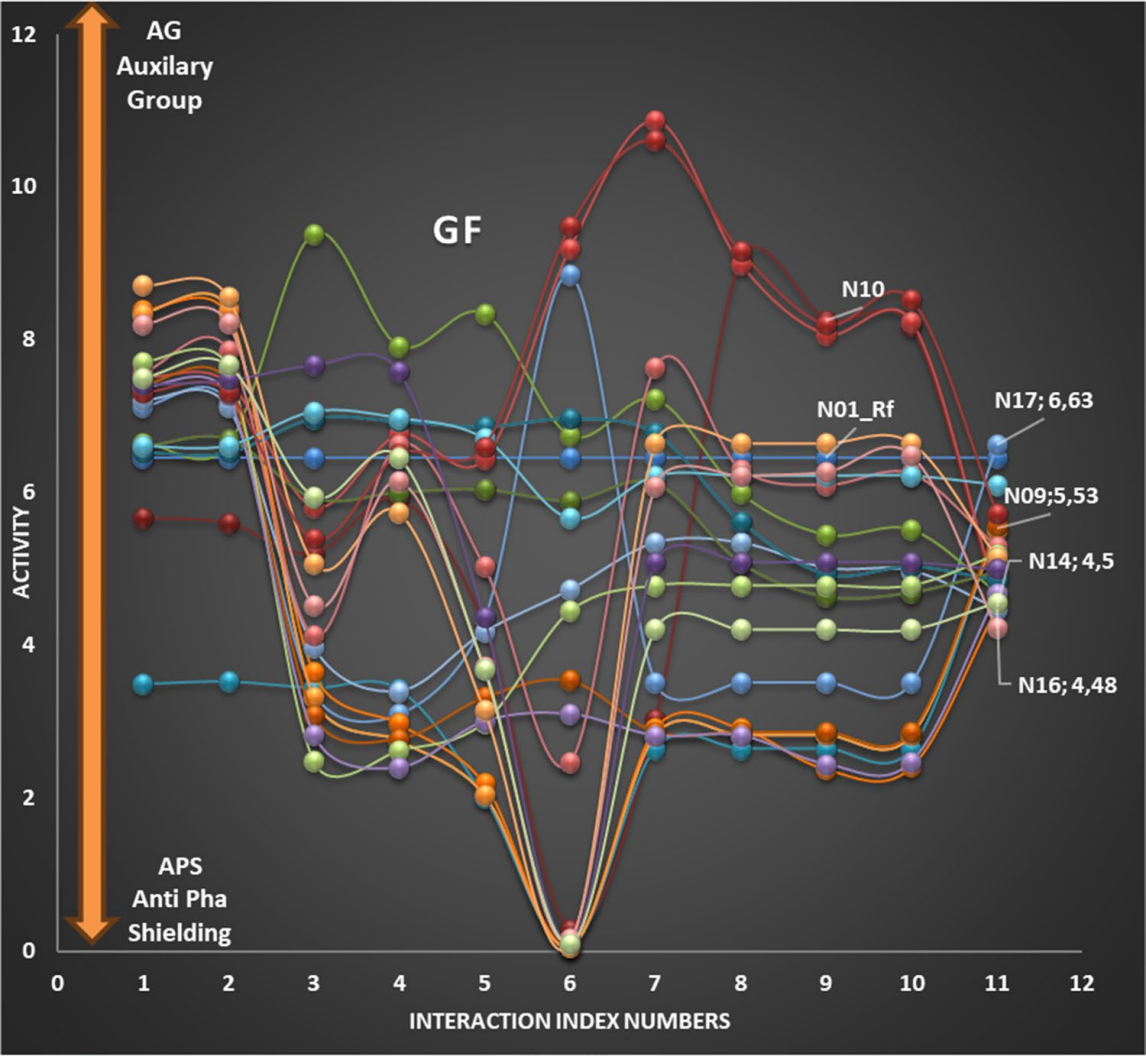
‘Graphical Fingerprint (GF) of a few randomly selected molecules from the investigated molecules

In the study, GF was observed at each interaction point for the series of molecules analyzed, and the changes in increasing (AG) and decreasing (APS) activity at *P* = 11 interaction points were similar and clearly visible in the graph lines depicted in Fig. 5. As shown by the arrows, AG and APS are examples of two separate points. The high and comparable statistical values obtained in the training and test sets, together with the internal validation showing similarity in GF values, attest to the stability and robustness of the model. The similarity of GF changes between the training set and the test set indicates that the model works effectively at each interaction point [71]. To ensure that the chosen model was not selected by chance, the GF validation of each point in both sets was conducted, indicating that the model is highly predictive and robust. Due to the large number of molecules analyzed, GF values for all molecules were not reported, and only some were shown. Molecules with very similar GF curves can be optimally subdivided in both sets.

The study observed that molecules with similar LRD values corresponding to one point of pharmacophore showed similar GF changes in activity. However, it is not possible for the LRDs of two molecules to be exactly the same at all points of interaction, and differences in activities between the two molecules can arise from either difference in LRD values or differences in geometric structures. Having a diverse set of molecules with different LRD and geometric structures is important for the development of the model. Models based on molecules with the same basic skeleton formed with the same geometric and LRD structures may not be sufficient to predict the activities of molecules with different basic skeletons. The study found that both the geometric and electronic differences of molecules with different basic skeletons often resulted in divergent GF lines, as shown in Fig. 5. The reliable model was developed from different independent variables provided by different skeleton molecules in the training set. Finally, it is worth noting that most (4/5) of the molecules with very similar GFs were included in the training set and only some (1/5) in the test set.

The robustness of the developed model, which was created by optimally dividing molecules between the training and test sets based on their GFs, indicates its high predictive ability. This means that a molecule with a GF similar to those in the model can be reliably evaluated in an external test set that was not used in the model fitting phase.

GF is a valuable tool in understanding the relative binding contributions of atoms at the point of interaction, including positive contributions from AG and negative contributions from APS. This information is crucial for computer-aided rational design of bioactive molecules and can help researchers visualize the skeletal structure and atom types of a new molecule. The GF also allows for easy comparison of molecules in the training and test sets, providing further evidence of the robustness and validity of the model.

The ongoing research on the development of pharmacophore with MCET not only identifies the fragments responsible for binding, but also measures their relative binding contribution at each interaction point. The GF-important binding sites (both positive, AG and negative, APS) highlighted in Fig. 5 provide crucial information for computer-aided rational design of bioactive molecules and visual analysis by researchers. Additionally, the GF analysis demonstrates how well the established model aligns with experimental reality.

The 3D QSAR model developed using MCET has the potential to explain the stereo configuration and structural modifications of molecules in terms of their observed binding affinities, measured as K_i_ values for the 26 fenoterol analogs. The findings of the model are partly consistent with those of previous studies [54, 72, 73]. According to the model, the β2-AR selectivity of fenoterol analogs is due to the adrenaline-like structure of the amino alkyl group located within the transmembrane (TM) components of the molecules. The model suggests that hydrogen bond interactions are formed between the p- and m-oxygen moieties on the phenyl ring of both fenoterol and methoxy phenoterol and tyrosine 308 (Y308) in TM7 and/or histidine 296 (H296) in TM6, contributing to the binding affinity.

In the developed 3D QSAR model using MCET, all the atoms in pharmacophore have been shown to contribute to the binding affinity through both non-bonding covalent interactions and electrostatic interactions based on HSAB theory. Specifically, the model identified that the selectivity of fenoterol analogs towards β2-AR is due to the adrenaline-like structure of the amino alkyl part of the molecules within the transmembrane (TM) components. The model also revealed that hydrogen bond interactions are formed between the p- and m-oxygen moieties on the phenyl ring in both fenoterol and methoxyphenoterol, and tyrosine 308 (Y308) in TM7 and/or histidine 296 (H296) in TM6. Additionally, the interactions of other C-atoms in the phenyl group are also included in the model. Notably, C-atoms 3 and 6 have a sterically adverse effect on the receptor with APS, while C-atom 7 has a highly electrostatic effect with AG, as shown in Fig. 5. Overall, the developed 3D QSAR model has the potential to explain the stereo configuration and structural modifications of fenoterol analogs and is partially consistent with previous studies.

## Conclusion

In this study, 3D-QSAR studies were conducted for 26 compounds of fenoterol stereoisomers that are effective on the β2AR target. As a result of computational studies, the Q^2^ value of this derivative set was calculated as 0.981 and the R^2^_ext_ value was calculated as 0.998, according to the Klopman (Electrostatic Lumo Klopman) descriptor. The fact that the results are greater than 0.9 indicates that a good model has been proposed in 3D-QSAR. Additionally, this study contributes to the literature. First, in cases where 3D-QSAR regression problems cannot distinguish stereo isomers, the clustering of molecules in the ZMR system has proven its usability by giving good results for healthy data sets. The second is to account for activity changes using GF interaction points. What this means is that it has been proven that latent, significant and low-dimensional GF can enable the prediction of experimentally measured or unmeasured molecular properties without the need for multidimensional analysis. The GF method used in this study offers many unique and innovative advantages. First, it allows 3D-QSAR predictive models to be understood and implemented graphically. Second, it allows the determination of APS and AG dimensions for each interaction domain of the model. Third, it predicts the most efficient atom types and AG values for an interaction point. Fourth, it serves as a safe tool to use GF as a reference in a molecular database created through quantum chemical calculations. Finally, it facilitates the interpretation of activity results, and GF analysis can help select the simplest and most active molecule.

## Data Availability

Availability of data and material. The data generated during and/or analyzed during the current study are available from the corresponding author on reasonable request.
